# Temporary Left Ventricular Unloading With Impella as a Bridge to Emergency Surgery for Acute Mitral Bioprosthetic Failure

**DOI:** 10.7759/cureus.116041

**Published:** 2026-09-10

**Authors:** Osamu Kinoshita, Takuma Aoki, Satoru Murata, Kento Murohara, Wataru Iai

**Affiliations:** 1 Department of Cardiovascular Surgery, National Hospital Organization Saitama Hospital, Wako, JPN; 2 Department of Cardiology, National Hospital Organization Saitama Hospital, Wako, JPN

**Keywords:** acute mitral regurgitation, bridge to surgery, cardiogenic pulmonary edema, impella, left ventricular unloading, mitral valve replacement, structural valve deterioration

## Abstract

Structural valve deterioration usually declares itself gradually, but a bioprosthetic leaflet that tears away from a stent post can produce catastrophic regurgitation without warning. In the mitral position, the left atrium has not had time to become compliant, and the resulting rise in pulmonary capillary pressure can flood the lungs within hours.

A 68-year-old woman who had undergone mitral valve replacement with a bovine pericardial bioprosthesis 13 years earlier developed sudden dyspnea. Her oxygen saturation was 58%, the chest radiograph showed almost complete bilateral opacification, and the pH was 7.042 with a lactate of 8.8 mmol/L. She was intubated after noninvasive ventilation failed. Catheterization excluded coronary disease and showed a cardiac index of 1.32 L/minute/m², a mean pulmonary artery pressure of 35 mmHg, and a wedge pressure of 22 mmHg. Echocardiography demonstrated severe regurgitation through the prosthesis with an abnormally mobile leaflet. Emergency surgery was indicated, but the operating room was occupied, and a wait of several hours was expected. With the pulmonary artery pressure rising, an Impella CP (Abiomed, Danvers, MA) was implanted approximately 11 hours after arrival and supported the circulation for five hours until cardiopulmonary bypass was established. During that interval, the rise in pulmonary artery pressure did not continue; the cardiac index and mixed venous oxygen saturation increased, and oxygenation was maintained despite a reduction in the fraction of inspired oxygen. Mean pulmonary artery pressure nevertheless remained approximately 40 mmHg.

One leaflet was found torn away from a stent post and prolapsing into the left atrium; the valve was replaced with a porcine bioprosthesis. Postoperatively, she required hemodiafiltration for acute kidney injury and temporary pacing for bradyarrhythmia; the course was further complicated by small cerebral infarcts and a right phrenic nerve palsy. She was extubated on day 10 and discharged home on day 48, remaining in New York Heart Association class I at review on day 108.

When definitive surgery for acute left-sided valvular failure must be delayed for logistical reasons, temporary left ventricular unloading may be considered as a means of protecting the lung during the wait. In this patient, it was associated with maintained oxygenation and improved systemic hemodynamics, although its independent contribution cannot be determined from a single case.

## Introduction

The lung tolerates acute hydrostatic injury poorly. When pulmonary capillary pressure rises abruptly, the alveolar-capillary barrier fails mechanically rather than simply leaking, with disruption of the capillary endothelium and the alveolar epithelium [1]. Repeated or sustained elevation of pulmonary venous pressure is followed by remodeling of the alveolar-capillary membrane and the pulmonary microvasculature, and by persistent impairment of gas diffusion [2,3]. These changes are not readily reversible: they do not respond to fluid removal by ultrafiltration, and they may persist after heart transplantation despite restoration of normal hemodynamics [2].

This matters because the options for a failed lung are far more limited than for a failed heart or kidney. Durable mechanical support and hemodialysis can sustain those organs for years, whereas extracorporeal gas exchange is only a short-term measure and lung transplantation is not an option in acute cardiogenic shock. In a patient whose heart can be repaired surgically, the lung is therefore the organ most likely to determine whether the operation comes too late.

Acute severe mitral regurgitation can produce this sequence within hours. Because the left atrium has not had time to dilate and become compliant, the regurgitant volume is transmitted almost directly to the pulmonary capillary bed [4], and the abrupt rise in hydrostatic pressure produces interstitial and then alveolar flooding [5]. Most reported cases arise from ischemic papillary muscle or chordal rupture; abrupt failure of a mitral bioprosthesis is a less familiar cause. Left ventricular unloading as a bridge to mitral valve surgery has been reported almost exclusively in ischemic papillary muscle rupture, where the aim was to stabilize circulation rather than protect the lungs [6-8]. We report a patient in whom a bioprosthetic leaflet tore away from a stent post 13 years after implantation. Emergency surgery was indicated seven hours after arrival, but the operating room did not become free for a further eight hours, and an Impella CP (Abiomed, Danvers, MA) was used during the second half of that interval with the explicit aim of limiting further pulmonary injury.

## Case presentation

History and presentation

A 68-year-old woman (height 151 cm, weight 39 kg) had undergone mitral valve replacement with a 27-mm bovine pericardial bioprosthesis (Carpentier-Edwards PERIMOUNT Magna Ease Mitral, Edwards Lifesciences, Irvine, CA) and tricuspid annuloplasty (MC³ 26 mm, Edwards Lifesciences) through a right minithoracotomy 13 years earlier, at the age of 55. She had hypertension, hyperuricemia, depression, and chronic kidney disease, and was a current smoker. Chest radiography and computed tomography (CT) performed 3.5 months earlier during surveillance for an unrelated condition showed well-aerated lung fields and served as a baseline (Figures 1A, 2A); pulmonary function testing at the same time demonstrated a mild restrictive defect without airflow obstruction.

**Figure 1:**
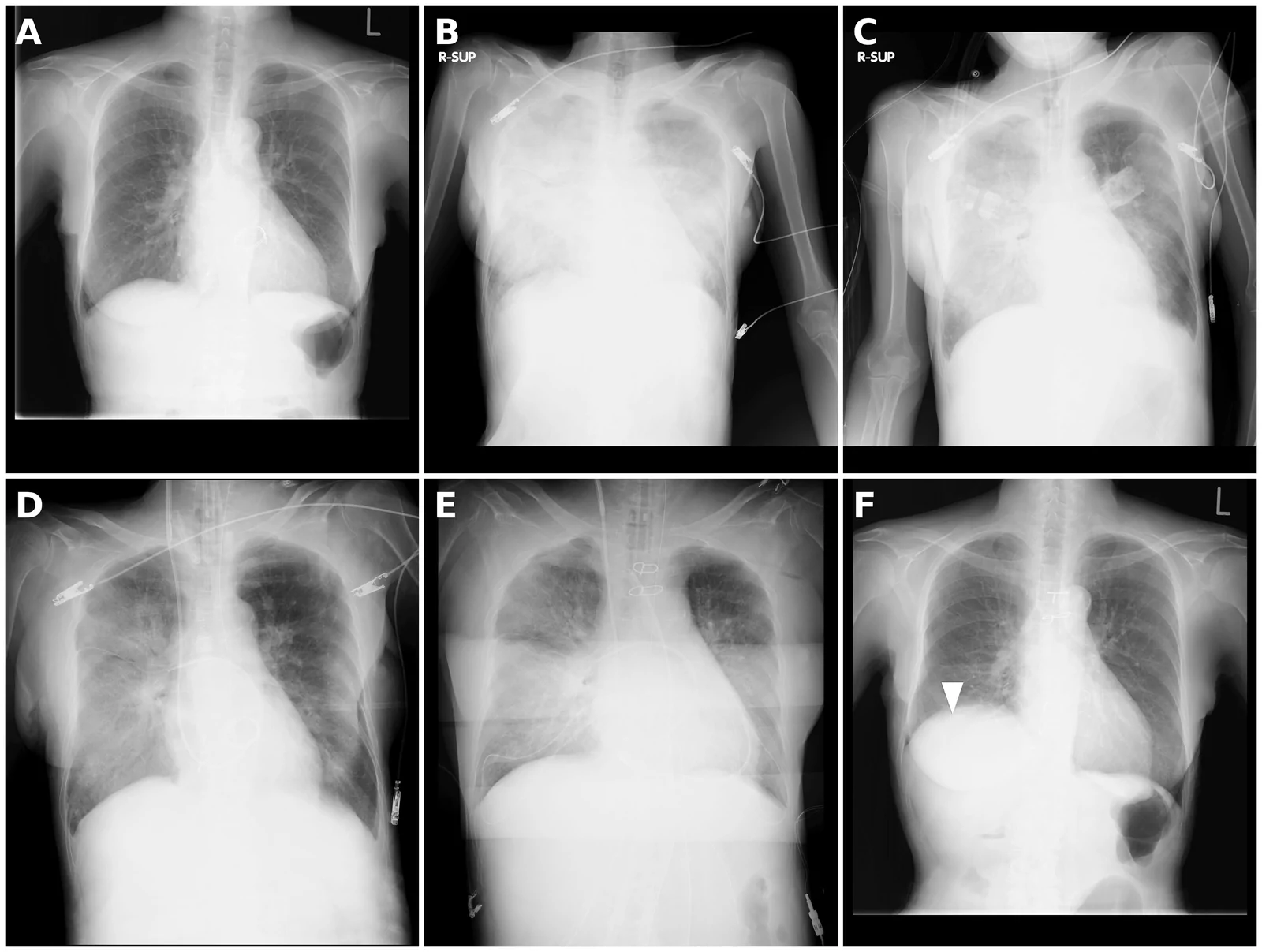
Serial chest radiographs from baseline to late follow-up (A) Baseline film obtained 3.5 months before admission, showing clear lung fields with the mitral prosthesis and the tricuspid annuloplasty ring. (B) Film obtained on arrival in the emergency department, showing near-complete opacification of both lung fields with loss of the cardiac silhouette. (C) After tracheal intubation. (D) Shortly before insertion of the Impella CP; the pulmonary artery catheter is in place. The image was cropped above the shoulders so that a positioning annotation lay outside the frame. (E) Film obtained at the end of the operation, showing a marked increase in aeration. (F) Day 73, in the outpatient clinic: the lung fields are clear, and the right hemidiaphragm remains elevated (arrowhead), indicating the right phrenic nerve palsy, which was not apparent while the patient was ventilated. (A) and (F) were obtained with the patient standing, and (B)-(E) with the patient supine, so diaphragmatic position cannot be compared directly across all panels These are clinical images exported from the original imaging data, to which panel letters and an arrowhead are added, marking regions of interest. Nothing in the figure is obscured or reconstructed, and no part of any image has been filled in or interpolated. The image was not generated or altered by generative artificial intelligence

**Figure 2:**
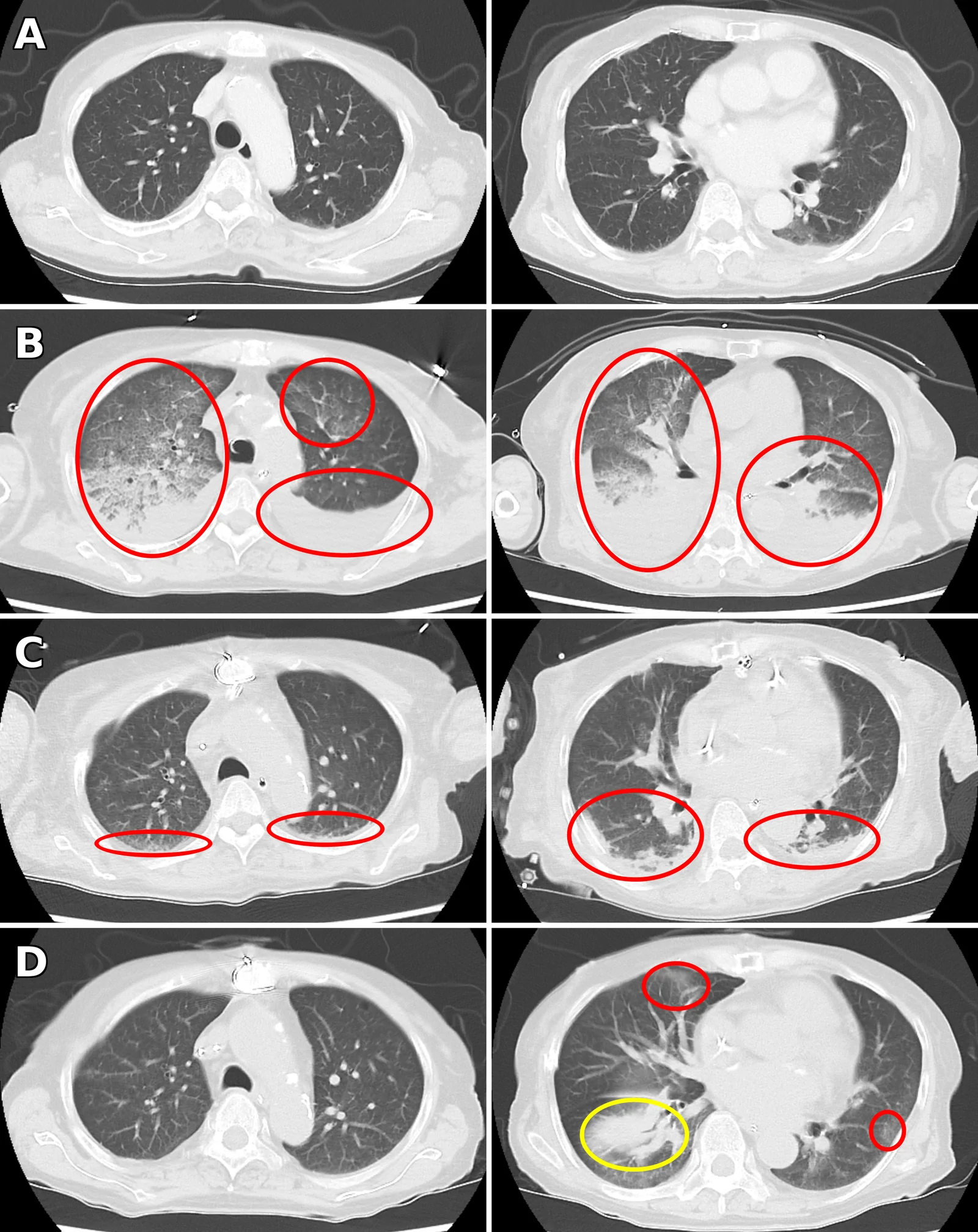
Serial computed tomography of the chest (lung window) Matched axial sections at two levels in every study: (Left) the middle of the fourth thoracic vertebra and (Right) the middle of the seventh thoracic vertebra. (A) 3.5 months before admission: well-aerated lung fields. (B) Day 0, shortly after intubation: diffuse bilateral ground-glass opacity with dependent consolidation and bilateral pleural effusions (red circles). (C) Day 3: markedly improved compared with (B), with only residual dependent consolidation and small pleural effusions (red circles). (D) Day 28: near-complete resolution, with residual right lower lobe atelectasis (yellow circle) and slight residual consolidation in each lung (red circles) These are clinical images exported from the original imaging data, to which panel letters and circles are added, marking regions of interest. Nothing in the figure is obscured or reconstructed, and no part of any image has been filled in or interpolated. The image was not generated or altered by generative artificial intelligence

Two hours before arrival, she developed sudden dyspnea and chest pain. When the emergency crew arrived, she was alert but tachypneic, with a systolic blood pressure of 254 mmHg, a heart rate of 120 beats/minute, and an oxygen saturation of 58% on room air. Oxygen was administered via mask during transport, and her level of consciousness declined.

Emergency department course

On arrival, she was tachypneic at 30 breaths/minute with an oxygen saturation of 62% on a reservoir mask, and her blood pressure was 194/115 mmHg (Table 1). There was marked wheezing throughout both lung fields, which obscured the heart sounds. The chest radiograph showed near-complete opacification of both lung fields with loss of the cardiac silhouette (Figure 1B), and the electrocardiogram demonstrated sinus tachycardia without ST-segment deviation. Arterial blood gas analysis shortly after arrival revealed a mixed respiratory and high-anion-gap metabolic acidosis, with a pH of 7.042 and a lactate of 8.8 mmol/L (Table 1). Transthoracic echocardiography performed by the attending cardiologist suggested prosthetic valve dysfunction: there was turbulent mosaic inflow from the left atrium directed toward the interventricular septum, with an eccentric jet of severe mitral regurgitation. The left ventricle was hyperkinetic without regional asynergy, with the ejection fraction visually exceeding 60%; the right-sided chambers were not enlarged, and the inferior vena cava was dilated with reduced respiratory variation. No images were archived, and no findings were recorded for the other valves.

**Table 1:** Vital signs, arterial blood gas analysis, and laboratory results on arrival at the emergency department Vital signs and blood samples were both obtained on arrival, about two hours before the day designated day 0. Reference ranges are those of the reporting laboratory, except for the vital signs and the estimated glomerular filtration rate, for which conventional adult values are given. Oxygen was being given by reservoir mask, so the fraction of inspired oxygen could not be determined and no PaO₂/FiO₂ ratio is reported; the reference value shown for pulse oximetry applies to room air PaCO₂: partial pressure of carbon dioxide; PaO₂: partial pressure of oxygen

Category	Variable	Value	Reference range
Vital signs	Body temperature, °C	36.0	36.1-37.2
	Heart rate, beats/minute	125	60-100
	Blood pressure, mmHg	194/115	90-140/60-90
	Respiratory rate, breaths/minute	30	12-20
	Oxygen saturation by pulse oximetry, %	62	≥95
Blood gas	pH	7.042	7.350-7.450
	PaCO₂, mmHg	83.2	35.0-45.0
	PaO₂, mmHg	50.3	80.0-100.0
	Arterial oxygen saturation, %	59.1	>95.0
	Bicarbonate, mmol/L	22.1	20.0-26.0
	Base excess, mmol/L	-9.7	-2.0 to +2.0
	Anion gap, mmol/L	25.6	10.0-18.0
	Lactate, mmol/L	8.8	0.5-2.0
	Glucose, mg/dL	418	70-109
Hematology	White blood cell count, ×10³/µL	19.1	3.3-8.6
	Hemoglobin, g/dL	10.8	11.6-14.8
	Hematocrit, %	35.1	35.1-44.4
	Platelet count, ×10³/µL	225	158-348
Coagulation	Prothrombin time-international normalized ratio	0.88	0.90-1.13
	Activated partial thromboplastin time, seconds	25.8	24.0-34.0
	D-dimer, µg/mL	2.8	≤1.0
Chemistry	Total protein, g/dL	7.7	6.6-8.1
	Albumin, g/dL	3.6	4.1-5.1
	Total bilirubin, mg/dL	0.7	0.4-1.5
	Aspartate aminotransferase, U/L	54	13-30
	Alanine aminotransferase, U/L	17	7-23
	Lactate dehydrogenase, U/L	503	124-222
	Alkaline phosphatase, U/L	102	38-113
	γ-glutamyl transferase, U/L	51	9-32
	Creatine kinase, U/L	144	41-153
	Creatine kinase-MB, U/L	19	4-21
	Blood urea nitrogen, mg/dL	20.5	8.0-20.0
	Creatinine, mg/dL	1.07	0.46-0.79
	Estimated glomerular filtration rate, mL/minute/1.73 m²	39.7	≥60
	Uric acid, mg/dL	3.0	2.6-5.5
	Sodium, mmol/L	140	138-145
	Potassium, mmol/L	3.5	3.6-4.8
	Chloride, mmol/L	97	101-108
	Calcium, mg/dL	8.4	8.8-10.1
	Inorganic phosphate, mg/dL	6.2	2.7-4.6
	C-reactive protein, mg/dL	0.15	0.00-0.14
Cardiac markers	Troponin I, pg/mL	19.6	<18.3
	N-terminal pro-B-type natriuretic peptide, pg/mL	4,369.8	≤125

Noninvasive ventilation at a fraction of inspired oxygen of 1.0, an intravenous vasodilator infusion, and a loop diuretic did not arrest the deterioration; she was intubated two hours after arrival (Figure 1C). She was admitted to the intensive care unit; this day is designated day 0. CT performed shortly afterward showed diffuse bilateral ground-glass opacity with dependent consolidation and bilateral pleural effusions (Figure 2B).

Catheterization

Cardiac catheterization, performed between three and five hours after arrival, showed normal coronary arteries, marked pulmonary venous hypertension, and a severely reduced cardiac index (Table 2). A pulmonary artery catheter was left in place. She returned to the intensive care unit ventilated, sedated, and receiving a loop diuretic; the recorded plan was semiurgent surgical re-replacement, and mechanical circulatory support was not considered at that stage. No inotrope or vasopressor was given before the operation, and the systemic pressure was never low.

**Table 2:** Hemodynamic data at the initial catheterization, at the repeat catheterization on day 17, and during pulmonary artery catheter monitoring on day 20 Reference ranges are conventional adult values; the catheterization reports do not state them. The day 0 measurements were made during the catheterization performed between three and five hours after arrival. Systemic arterial pressure was measured in the aorta on days 0 and 17 and in the radial artery on day 20. Cardiac output was measured by the Fick method on day 0, by both methods on day 17, and by thermodilution through the pulmonary artery catheter on day 20. The two methods yielded substantially different values on day 17, and values derived from each are therefore reported; the day 17 and day 20 thermodilution measurements can be compared directly. The day 20 values were recorded through the pulmonary artery catheter and an indwelling radial arterial line shortly before the catheter was removed. The transpulmonary pressure gradient and the pulmonary vascular resistance were calculated from the values in the table

Variable	Day 0	Day 17	Day 20	Reference range
Heart rate, beats/minute	107	74	65	60-100
Right atrial pressure, mean, mmHg	11	9	2	0-8
Right ventricular pressure (systolic/end-diastolic), mmHg	45/13	41/10	-	15-30/1-7
Pulmonary artery pressure (systolic/diastolic/mean), mmHg	45/21/35	43/13/25	26/8/15	15-30/4-12/9-18
Pulmonary capillary wedge pressure (a/v/mean), mmHg	27/24/22	22/15/14	-	4-12 (mean)
Left ventricular pressure (systolic/end-diastolic), mmHg	111/16	171/14	-	90-140/5-12
Systemic arterial pressure (systolic/diastolic/mean), mmHg	120/78/97	155/63/99	120/38/62	90-140/60-90/70-105
Cardiac output (Fick), L/minute	1.75	3.12	-	-
Cardiac output (thermodilution), L/minute	-	5.35	3.2	-
Cardiac index (Fick), L/minute/m²	1.32	2.35	-	2.5-4.0
Cardiac index (thermodilution), L/minute/m²	-	4.02	2.6	2.5-4.0
Transpulmonary pressure gradient, mmHg	13	11	-	<12
Pulmonary vascular resistance (Fick), Wood units	7.4	3.5	-	<2
Pulmonary vascular resistance (thermodilution), Wood units	-	2.1	-	<2

Decision to use temporary circulatory support

The cardiac surgical team, engaged in another cardiovascular emergency, was consulted seven hours after arrival, at the end of that operation. Bedside transthoracic echocardiography, performed by the attending cardiologist at the consultation, showed severe regurgitation through the prosthesis and an abnormally mobile leaflet, with left ventricular function unchanged from the study on arrival. It was a brief screening examination, and no images were archived. The team judged that emergency mitral valve re-replacement was required. A further emergency operation and the reprocessing of instruments then intervened, and several more hours were expected to pass before she could be taken to the operating room.

Over the ensuing hours, the mean pulmonary artery pressure fluctuated between 33 and 48 mmHg, the cardiac index, by thermodilution, was between 1.7 and 2.0 L/minute/m², and the mixed venous oxygen saturation was between 46% and 52%. Nine hours after arrival, the systolic pulmonary artery pressure rose to 62 mmHg (mean, 46 mmHg). Faced with a wait of several more hours in a patient with already severely injured lungs, we asked our cardiology colleagues to insert an Impella CP for left ventricular unloading, specifically to limit further pulmonary congestion and support cardiac output during that interval.

The Impella CP was implanted through the left femoral artery approximately 11 hours after arrival; Figure 1D shows the radiograph obtained shortly before implantation. The pump was operated at performance level P-9, with a mean flow of 3.3 L/minute, and supported circulation for five hours. No clinically evident hemolysis, limb ischemia, major bleeding, or device-related vascular complication occurred during support.

Interval on support

Arterial oxygenation was maintained while the fraction of inspired oxygen was reduced from 0.50 to 0.40; the ratio of arterial oxygen tension to the fraction of inspired oxygen (PaO₂/FiO₂) was 325 immediately before implantation and 335 afterward, and lactate remained at 1.6 mmol/L. The cardiac index, previously 1.7-2.0 L/minute/m², rose to 2.3, and mixed venous oxygen saturation, previously 46%-52%, rose to 58%. Mean arterial pressure was 79-110 mmHg before implantation and 114-126 mmHg while the pump was running. Urine output was maintained.

After implantation, the rise in pulmonary artery pressure did not continue, although the mean pressure did not fall substantially: compared with the 62/32 mmHg (mean, 46 mmHg) recorded before implantation, the pressure immediately before transfer to the operating room was 58/24 mmHg (mean, 40 mmHg), with a central venous pressure of 9 mmHg. The course of oxygenation and hemodynamics over the first 12 days is shown in Figure 3.

**Figure 3:**
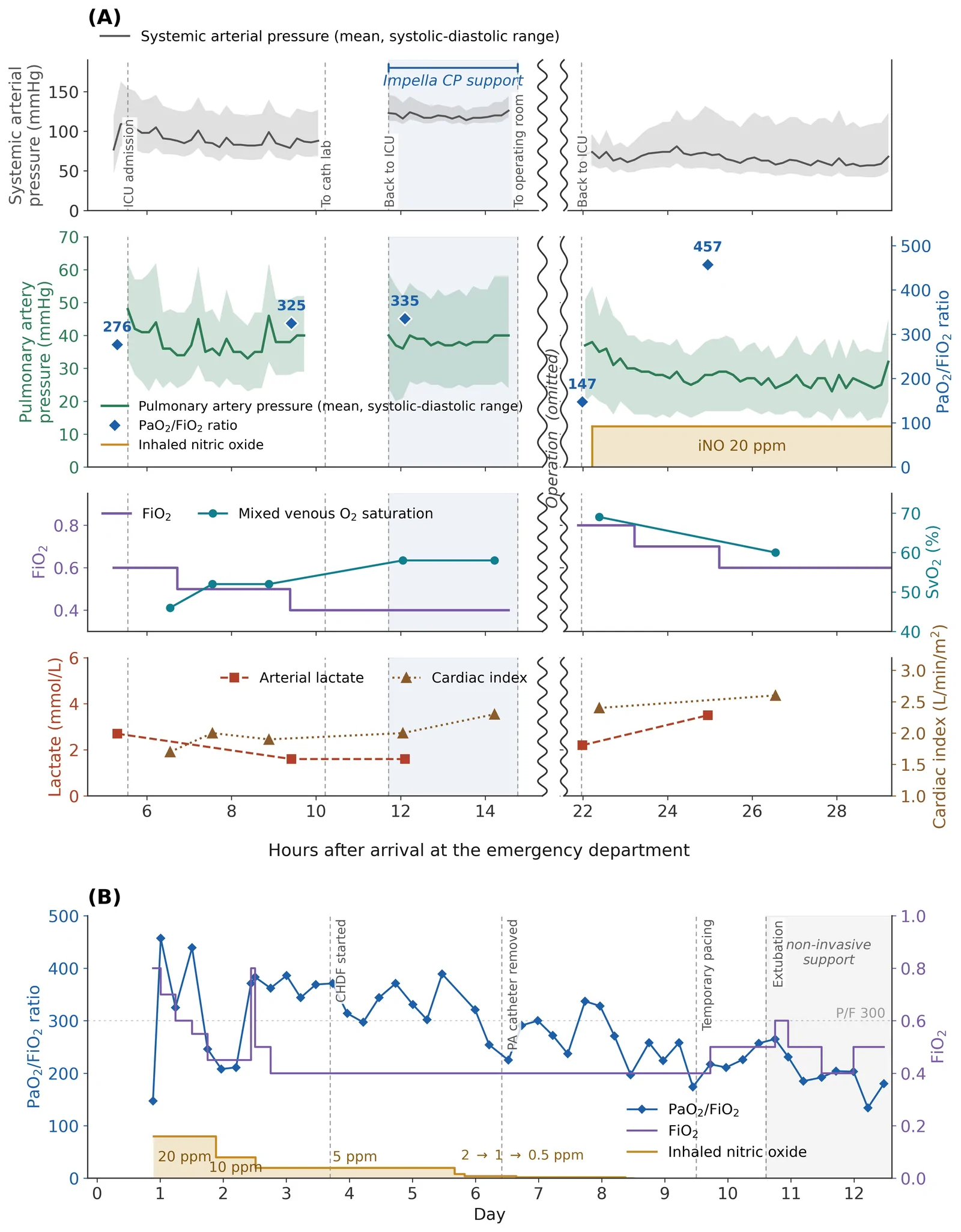
Time course of oxygenation and hemodynamics (A) From admission to the intensive care unit until approximately 29 hours after arrival, shown as four stacked panels with a common time axis: systemic arterial pressure; mean pulmonary artery pressure with the PaO₂/FiO₂ ratio; the fraction of inspired oxygen with mixed venous oxygen saturation; and arterial lactate with the cardiac index. The horizontal bracket marks the interval of Impella CP support, and the wavy break marks the operation, during which intensive care unit measurements are not available. (B) Days 0-12, showing the PaO₂/FiO₂ ratio and the fraction of inspired oxygen; the shaded band marks the period after extubation. Shaded ribbons in both panels represent inhaled nitric oxide and are schematic, with the dose annotated in ppm PaO₂: partial pressure of oxygen; FiO₂: fraction of inspired oxygen; iNO: inhaled nitric oxide; SvO₂: mixed venous oxygen saturation; CHDF: continuous hemodiafiltration; PA: pulmonary artery; P/F: partial pressure of oxygen/fraction of inspired oxygen This image was created by the authors from the patient’s clinical record using Python 3 (Python Software Foundation, San Francisco, CA) with the Matplotlib plotting library (Matplotlib Development Team, United States). The image was not generated or altered by generative artificial intelligence

Operation

She was taken to the operating room 15 hours after arrival. Transesophageal echocardiography after induction of anesthesia, with the Impella CP running, showed only mild-to-moderate mitral regurgitation, in contrast to the severe regurgitation seen before implantation (Figures 4A-4C).

**Figure 4:**
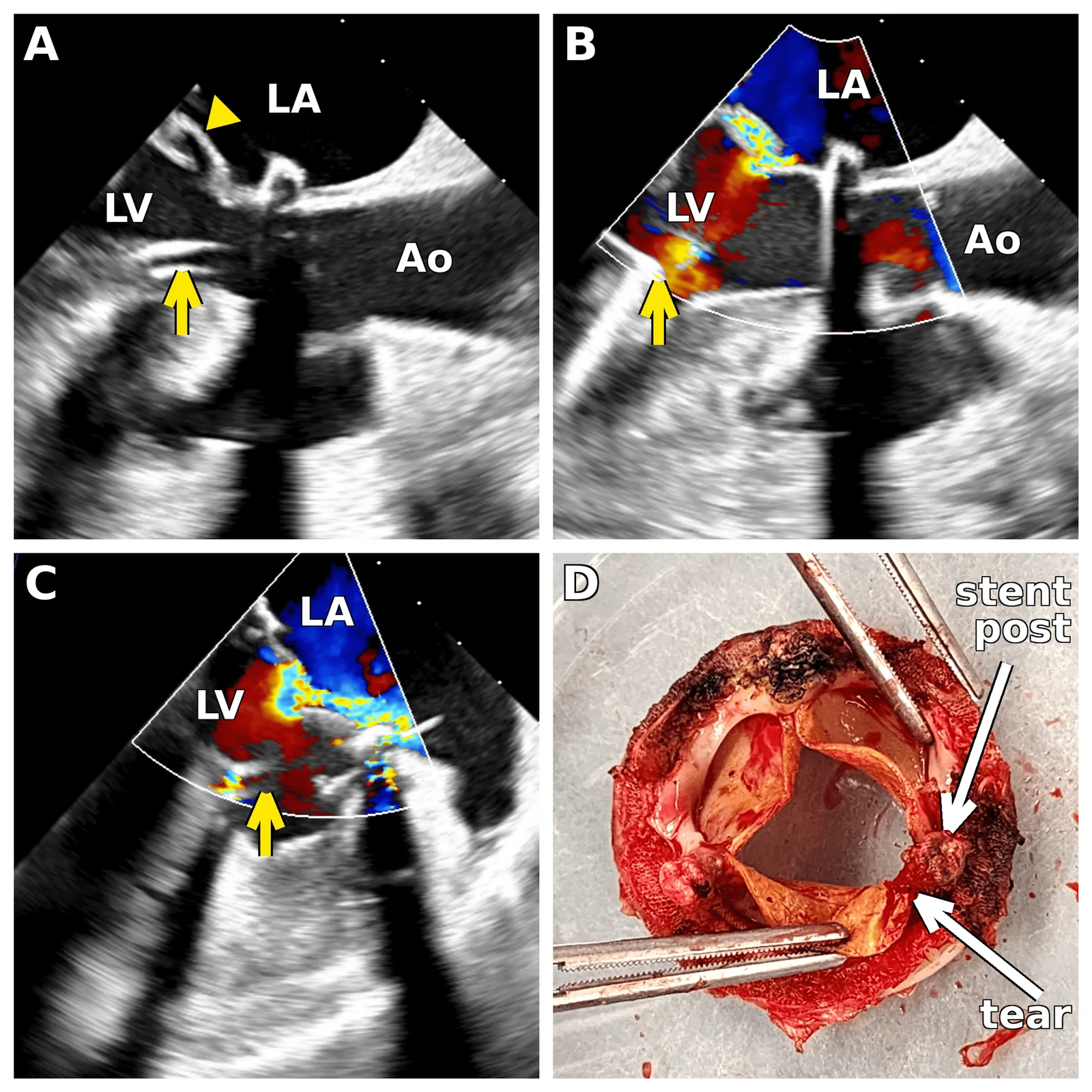
Mechanism of acute bioprosthetic valve failure (A) Intraoperative transesophageal echocardiography, mid-esophageal long-axis view. A prosthetic leaflet has detached and prolapsed into the left atrium (yellow arrowhead). (B) A comparable long-axis view with color Doppler. (C) Mid-esophageal commissural view with color Doppler. Yellow arrows in (A)-(C) indicate the Impella CP catheter within the left ventricle. (D) The explanted bioprosthesis viewed from the ventricular side; the white arrows indicate the stent post and the site of the tear. Brightness and contrast were adjusted uniformly across the whole photograph Ao: aorta; LA: left atrium; LV: left ventricle Images A-C are clinical images exported from the original imaging data; image D is a photograph of the explanted bioprosthesis. Panel letters, arrows, and an arrowhead are added, marking regions of interest. Nothing in the figure is obscured or reconstructed, and no part of any image has been filled in or interpolated. The image was not generated or altered by generative artificial intelligence

A median sternotomy was performed, and the left groin was opened to expose the Impella CP entry site. After cardiopulmonary bypass was established, the pump was withdrawn into the ascending aorta and removed. Through a right-sided left atriotomy, the left atrial appendage was closed from within in anticipation of postoperative atrial fibrillation; neither the atrium nor the appendage contained thrombus. The bioprosthesis showed no paravalvular dehiscence, and the annulus was free of calcification; the leaflets were thickened and sclerotic, but the valve was not appreciably stenotic, and on the posterior commissural side, one leaflet was torn away from a stent post and prolapsed into the left atrium (Figure 4D). Macroscopically, there was no vegetation, no pannus, and no thrombus; calcification was inconspicuous, and apart from the tear and the leaflet thickening, no other degenerative change was evident. A 27-mm porcine bioprosthesis (Epic Plus Mitral, Abbott, Abbott Park, IL) was implanted with pledgeted everting mattress sutures. Cardiopulmonary bypass time was 181 minutes and aortic cross-clamp time 113 minutes.

Postoperative course

She returned to the intensive care unit 22 hours after arrival, ventilated and receiving inotropic and vasodilator infusions; inhaled nitric oxide at 20 ppm was started shortly afterward. Mean pulmonary artery pressure fell from 40 mmHg preoperatively to 25-30 mmHg, the cardiac index rose to 2.4-2.9 L/minute/m², and mixed venous oxygen saturation rose to 56%-73%. The chest radiograph obtained at the end of the operation showed a marked increase in aeration (Figure 1E). Inhaled nitric oxide was weaned over the first postoperative week and stopped on day 8. Two sets of blood cultures obtained on day 2 yielded no growth.

Gas exchange improved more slowly (Figure 3B). The first postoperative blood gas, drawn before inhaled nitric oxide was started, showed a PaO₂/FiO₂ ratio of 147 at a fraction of inspired oxygen of 0.80. On day 1, the ratio ranged from 208 to 457, and on day 2, the chest radiograph showed increased bilateral effusions and edema, requiring higher positive end-expiratory pressure and a transient increase in the fraction of inspired oxygen. All 12 measurements made while inhaled nitric oxide was being given at 5 ppm exceeded 290 at a fraction of inspired oxygen of 0.40, whereas none of the 17 made after it was stopped did so (range, 134-265), and the fraction of inspired oxygen had to be raised again. CT on day 3 showed marked improvement compared with the study on admission (Figures 2B, 2C). She was extubated on day 10, but supplemental oxygen was required until day 23, with a step back from nasal cannula to high-flow oxygen between days 15 and 20. The course was also complicated by acute kidney injury requiring hemodiafiltration, small cerebral infarcts, infection, and transient atrial fibrillation and bradyarrhythmia requiring temporary ventricular pacing. A right phrenic nerve palsy, presumed to have been sustained during the operation, became apparent after extubation (Figure 5). The infarcts, in the left occipital lobe and the left cerebellar hemisphere, were detected by CT on day 6; the mechanism could not be established.

**Figure 5:**
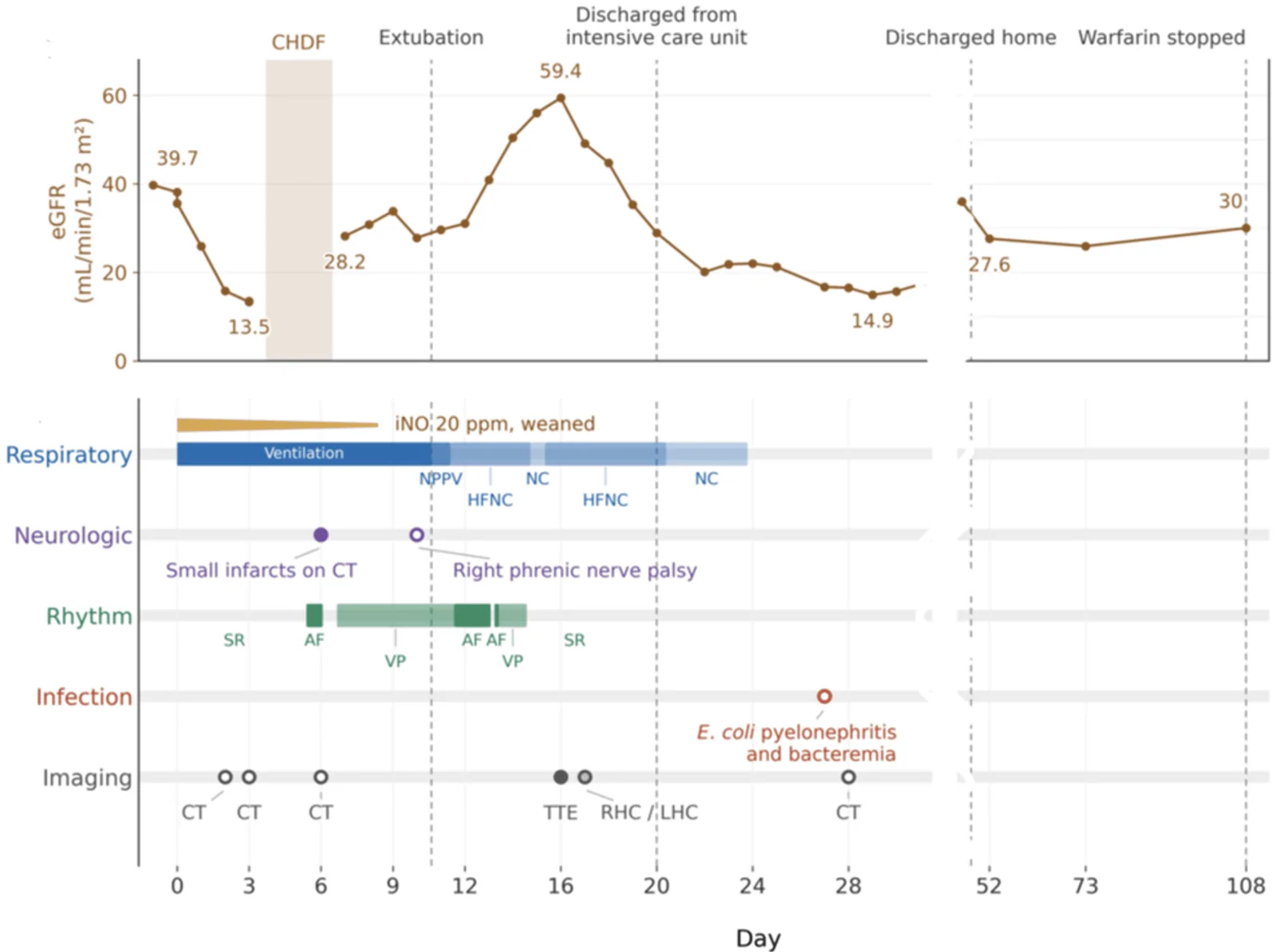
Postoperative course (Upper panel) Serial estimated glomerular filtration rate, calculated by the laboratory from the measured serum creatinine concentration. The shaded band marks continuous hemodiafiltration, during which values are not shown. (Lower panel) Events by organ system. On the respiratory row, each mode of delivery is shown in its own shade; the tapering band represents inhaled nitric oxide, weaned over the first postoperative week. On the rhythm row, the darker bars indicate atrial fibrillation and the paler bars indicate ventricular pacing; the remaining periods are sinus rhythm. On the imaging row, the circles are open for CT, filled for echocardiography, and gray for catheterization; on the neurologic row, they are filled for the infarcts and open for the phrenic nerve palsy. Vertical dashed lines mark extubation, discharge from the intensive care unit, discharge home, and cessation of warfarin. The horizontal axis is broken between days 31 and 45, and the right-hand segment is compressed AF: atrial fibrillation; CHDF: continuous hemodiafiltration; CT: computed tomography; eGFR: estimated glomerular filtration rate; HFNC: high-flow nasal cannula; iNO: inhaled nitric oxide; LHC: left heart catheterization; NC: nasal cannula; NPPV: noninvasive positive-pressure ventilation; RHC: right heart catheterization; SR: sinus rhythm; TTE: transthoracic echocardiography; VP: ventricular pacing This image was created by the authors from the patient’s clinical record using Python 3 (Python Software Foundation, San Francisco, CA) with the Matplotlib plotting library (Matplotlib Development Team, United States). The image was not generated or altered by generative artificial intelligence

Transthoracic echocardiography on day 16 showed a nondilated left ventricle with preserved systolic function, no appreciable regurgitation through the prosthesis (Figure 6), and a tricuspid regurgitant pressure gradient of 37 mmHg (Table 3). Tachypnea persisted. Because this gradient suggested residual pulmonary hypertension even though her weight had returned to the preoperative level, right and left heart catheterization was repeated on day 17. The mean pulmonary artery pressure was 25 mmHg with a wedge pressure of 14 mmHg, the left ventricular end-diastolic pressure was 14 mmHg, and the systemic pressure was 155/63 mmHg (Table 2). Diuresis and antihypertensive treatment were intensified; by day 20, she had lost a further 3.5 kg, and the mean pulmonary artery pressure had fallen to 15 mmHg (Table 2).

**Figure 6:**
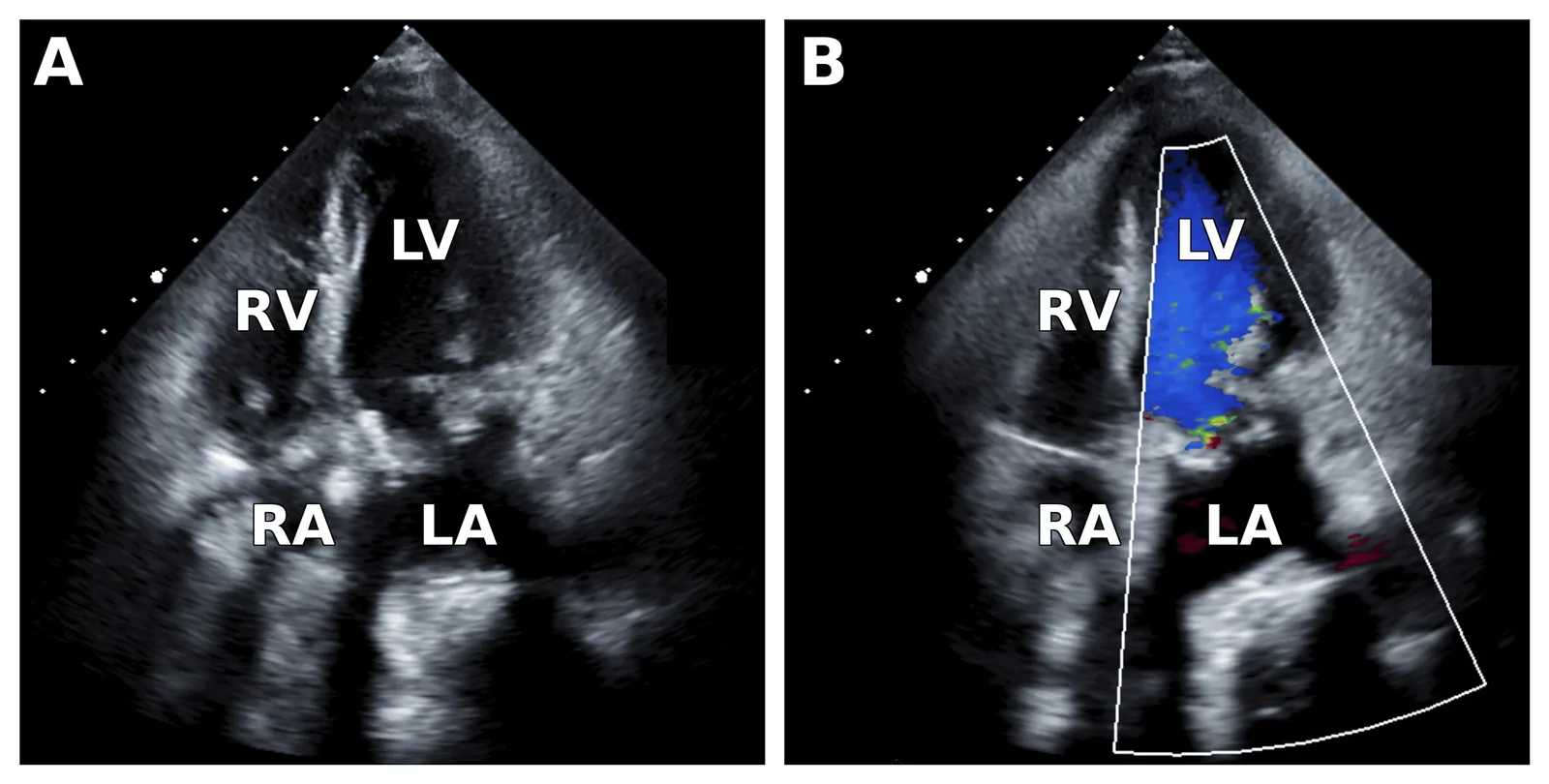
Transthoracic echocardiography on day 16 (A) Apical four-chamber view. (B) The same view in systole with color Doppler. No appreciable regurgitant jet is seen at the mitral prosthesis LA: left atrium; LV: left ventricle; RA: right atrium; RV: right ventricle Two machine-generated overlays in the black margin and the portion of the electrocardiographic trace intruding into the cropped field were painted out; neither lies in a diagnostically relevant region. These are clinical images exported from the original imaging data, to which panel letters are added. Nothing else in the figure is obscured or reconstructed, and no part of any image has been filled in or interpolated. The image was not generated or altered by generative artificial intelligence

**Table 3:** Transthoracic echocardiography on day 16 Reference ranges without a dagger are those printed on the report by the issuing laboratory. A dash indicates a measurement for which no reference value exists. The reference values for the mitral prosthesis are those conventionally taken to indicate normal function; they are flow-dependent, and the report itself noted that the mean gradient of 8 mmHg was elevated. The estimated right atrial pressure was 8 mmHg and the estimated right ventricular systolic pressure 45 mmHg. The left ventricle and the left atrium were of normal size, consistent with regurgitation of acute onset without antecedent chronic volume loading ^†^Conventional adult values, used where the laboratory does not define a reference value

Variable	Value	Reference range
Left ventricular end-diastolic diameter, mm	42	40-56
Left ventricular end-systolic diameter, mm	27	22-38
Interventricular septal thickness, mm	10	7-10
Posterior wall thickness, mm	10	7-10
Relative wall thickness	0.48	<0.42
Fractional shortening, %	35.7	27-45^†^
Ejection fraction (Teichholz), %	65.6	55-74
Left atrial diameter, mm	32	24-40
Left atrial volume index, mL/m²	31	10-34
Aortic root diameter, mm	24	22-36^†^
Mitral prosthesis, peak velocity, cm/s	209	<190^†^
Mitral prosthesis, mean velocity, cm/s	130	-
Mitral prosthesis, peak gradient, mmHg	18	-
Mitral prosthesis, mean gradient, mmHg	8	≤5^†^
Mitral prosthesis, velocity-time integral, cm	58.0	-
Mitral regurgitation	Trivial	None or trivial^†^
Tricuspid regurgitation	Trivial	None or trivial^†^
Tricuspid regurgitant peak velocity, cm/s	305	<280^†^
Tricuspid regurgitant pressure gradient, mmHg	37	<31^†^

CT on day 28 showed near-complete resolution of the pulmonary opacities, with only residual right lower lobe atelectasis and slight residual consolidation in each lung (Figure 2D). She did not require chronic renal replacement therapy and was discharged home on day 48. At review on days 52, 73, and 108, she was in New York Heart Association class I [9] and in sinus rhythm, and the chest radiograph showed no pulmonary congestion, with unchanged elevation of the right hemidiaphragm (Figure 1F). Renal impairment persisted, with an estimated glomerular filtration rate of 26-30 mL/minute/1.73 m². Warfarin was stopped on day 108, more than three months after bioprosthetic valve replacement.

## Discussion

Abrupt structural valve deterioration

Structural valve deterioration is usually a gradual degeneration of leaflet tissue, although sudden valve dysfunction has been described [10]. The standardized definitions grade deterioration by progressive changes in gradient or regurgitation on serial imaging, but they also state that an acute and large structural change in a leaflet may bypass the intermediate stages and constitute stage 3 deterioration and bioprosthetic valve failure at once [11]. This presentation is consistent with that exception.

Failure at 13 years is consistent with the reported timing: structural deterioration due to leaflet tear occurs at a mean of 14.8 years after implantation of a Carpentier-Edwards pericardial mitral bioprosthesis and accounts for 19.7% of all such deterioration [12]. What was unexpected was the manner of failure: the leaflet separated from a stent post, converting a functioning valve into a severely regurgitant one over minutes.

The closest reported case is that of a porcine mitral bioprosthesis in which a cusp separated at its base from the valve frame, without calcification or infection. That presentation was initially attributed to cardiogenic pulmonary edema, and the diagnosis was further confounded by concomitant pneumonia [10]. The present case differs in valve type and in the interval to failure, but the mode of failure is the same.

Why the lung was the priority

Elevated pulmonary capillary pressure does not merely produce transudation. Above a critical pressure, the capillary wall fails structurally, with breaks in the endothelium and the alveolar epithelium, and leakage of protein-rich fluid and red cells into the alveoli [1]; the clinical thresholds are a left atrial pressure of 18-25 mmHg for interstitial edema and one above 25 mmHg for alveolar flooding [5]. The wedge pressure of 22 mmHg was measured after intubation and diuresis and therefore probably underestimated the peak value.

Choice of device

The Impella CP is a transvalvular microaxial pump that draws blood directly from the left ventricle and ejects it into the ascending aorta, independently of the cardiac cycle. The pressure-volume loop is therefore shifted leftward; left ventricular end-diastolic volume and pressure decrease, and left atrial and pulmonary capillary wedge pressures fall with them [13]. The intra-aortic balloon pump also reduces afterload, and in acute mitral regurgitation, it reduces the regurgitant volume, making it a reasonable alternative; the ventricular function and stable rhythm on which its augmentation depends were both present here. But counterpulsation supports pressure rather than flow, and it has not been shown to reduce end-diastolic pressure and volume to the degree achieved by transvalvular unloading [13,14]. With the orifice fixed by a leaflet torn from a stent post, a cardiac index of 1.7-2.0 L/minute/m², and a mixed venous oxygen saturation of 46%-52%, we preferred a device that supplies forward flow independently of ventricular ejection. Both effects corresponded directly to the pathophysiology in this patient.

Venoarterial extracorporeal membrane oxygenation (VA-ECMO) increases left ventricular afterload, and the resulting rise in filling pressure can worsen pulmonary congestion and cause acute lung injury [13,14]; observational data suggest that concomitant unloading is associated with lower mortality [15-17]. In this patient, whose oxygenation was already adequate on mechanical ventilation and whose problem was pulmonary venous hypertension rather than systemic hypoxemia, direct left ventricular unloading was the more logical choice. The randomized evidence supporting the Impella CP is confined to infarct-related cardiogenic shock [18], and its use here was an extrapolation.

Impella support as a bridge to surgery in acute mitral regurgitation has been reported previously, almost always for ischemic papillary muscle rupture [6-8], sometimes with VA-ECMO [19]. Our case adds bioprosthetic leaflet tears to that list and differs mainly in the reason for the delay: the operation was deferred not for physiologic stabilization but because the operating room was unavailable. Transcatheter mitral valve-in-valve implantation is not approved in Japan; surgical re-replacement was therefore the only definitive option, and the wait was unavoidable.

Aspects of management that could have been improved

Systemic afterload is the most direct determinant of regurgitant volume, and therefore of left atrial and pulmonary capillary pressure, in acute mitral regurgitation. A vasodilator was used in the emergency department, but none was given after admission to the intensive care unit; thereafter, the only continuous cardiovascular infusion was a loop diuretic. While the ventricle was being mechanically unloaded, the load against which it ejected was allowed to rise, and this rise may be one reason the mean pulmonary artery pressure did not fall substantially during support. The later course in this patient is consistent with the importance of afterload and volume control: after the repeat catheterization on day 17, intensified antihypertensive treatment and diuresis lowered the mean pulmonary artery pressure from 25 to 15 mmHg over three days. The estimated glomerular filtration rate fell in parallel, from 49 mL/minute/1.73 m² on day 17 to 20 mL/min/1.73 m² on day 22 (Figure 5); the same volume-loaded state had supported both the pulmonary pressure and the filtration rate.

The second criticism concerns timing. Severe pulmonary congestion was documented at the diagnostic catheterization, which began three hours after arrival, when she was on the catheterization table, and femoral access could have been obtained without delay; the Impella CP was not inserted until eight hours later, and then only after it had become clear that the operating room would not be free. With hindsight, the moment to begin unloading in this patient was when the congestion was demonstrated, not when the delay became apparent. Whether earlier support would have altered the pulmonary course cannot be known from one patient, and the suggestion that it would have done so is speculative; the duration of the pressure elevation, however, and not only its peak, determines the residual injury: among patients with a first myocardial infarction and preserved ejection fraction, those in whom alveolar-capillary membrane conductance failed to recover within a week, and in whom wedge pressure subsequently rose, had acute alveolar edema within one year in 37% of cases, against 2.5% in those in whom it recovered [20].

How long should such a bridge last?

Intraoperative echocardiography after induction of anesthesia showed that the regurgitation had decreased from severe to mild-to-moderate. General anesthesia and positive-pressure ventilation reduce afterload and make mitral regurgitation appear less severe; the pump itself diverts part of the left ventricular volume away from the mitral orifice. No images were recorded immediately after implantation under comparable conditions, so the change can be neither quantified nor attributed to the pump. The mismatch was notable: the regurgitation had fallen substantially while the mean pulmonary artery pressure had not.

Her lungs recovered slowly. Although the chest radiograph improved strikingly within hours of valve replacement, oxygenation depended on inhaled nitric oxide for eight days, extubation was not possible until day 10, and supplemental oxygen was needed until day 23. Pulmonary hypertension also persisted. Mean pulmonary artery pressure was 46 mmHg before implantation; approximately 40 mmHg on support; 25-30 mmHg during the first postoperative days, on vasodilators and hemodiafiltration; 25 mmHg at catheterization on day 17, when the systemic pressure was also high; and 15 mmHg on day 20 (Table 2). The residual pulmonary hypertension was therefore hemodynamic rather than fixed, and to that extent, the pulmonary circulation escaped the remodeling that follows sustained elevation [2,3]. The two earlier periods were not managed in the same way: no vasodilator was given during support, whereas during the first postoperative days, vasodilators were combined with hemodiafiltration. Therefore, it cannot be determined when the pulmonary circulation became responsive. The hydrostatic insult itself is relieved within minutes, and the ultrastructural breaks in the alveolar-capillary barrier close rapidly once transmural pressure falls [1]; gas transfer recovers far more slowly, and pulmonary inflammation persists for days after the edema itself has cleared [2].

If the pump relieved the regurgitation and arrested the rise in pulmonary pressure, it is reasonable to ask whether the operation should have been deferred for several days to allow the lung to recover before exposure to cardiopulmonary bypass. Support of this kind has been reported as a bridge to mitral surgery for up to a week [6]. In those reports, the delay was intended to stabilize hemodynamics, to control infection, or to allow infarcted myocardium to mature [6,8,19]; in no case was it intended to allow an injured lung to recover.

We cannot say whether deferral would have been better, and there are substantial arguments against it. The lesion was a mechanical failure of a prosthesis, and its progression could neither be monitored nor predicted; had the torn leaflet separated further, the regurgitation would have become severe again despite support, in a patient with limited physiologic reserve. Microaxial pump support for several days is not benign [18]. Acute severe mitral regurgitation with cardiogenic shock is conventionally an indication for urgent operation, and deferral would be a departure from that standard. We raise the question not because we advocate deferral but because the answer determines what a device of this kind is for in this setting: whether it is a means of surviving the wait for an operating room, or a means of choosing when to operate.

Limitations

The contribution of the device cannot be isolated. Oxygenation had already improved substantially after intubation and diuresis, and the improvement in the PaO₂/FiO₂ ratio from 276 to 325 preceded implantation. Positive-pressure ventilation itself reduces both left ventricular preload and afterload, and relieves acute cardiogenic pulmonary edema; it was in place throughout the period of support, and its effect cannot be separated from that of the device. What can be said is narrower: during the five hours on support, gas exchange was maintained, with a reduction rather than an increase in the fraction of inspired oxygen; lactate remained normal; and the cardiac index and mixed venous oxygen saturation rose. The mean pulmonary artery pressure did not fall substantially, although the rise that had prompted the decision to implant the pump did not continue; the benefit that can be documented objectively is therefore systemic rather than pulmonary. Pulmonary capillary wedge pressure was not remeasured after implantation, and no chest imaging was obtained during support; therefore, a direct reduction in pulmonary venous pressure or pulmonary edema was not documented. Pulmonary hypertension was not abolished, and we do not claim that the device reversed it. The severity of the regurgitation before implantation was recorded only qualitatively. Whether the patient would have deteriorated without support is unknowable. Nor can one patient establish when unloading should begin: what is reported here is feasibility in a single case, not comparative evidence for a timing threshold.

The explanted valve was submitted for neither culture nor histopathological examination, and blood cultures were not obtained before the operation. The conclusion about the mechanism of failure therefore rests on the macroscopic appearance of the valve. Although the C-reactive protein concentration of 0.15 mg/dL on admission, the absence of vegetation, and the negative postoperative blood cultures make prosthetic valve endocarditis very unlikely, it was not excluded microbiologically.

The mechanism of the cerebral infarcts cannot be established. Head CT before the initial catheterization showed no acute lesion, and in retrospect, the infarcts were faintly visible on the head CT obtained on day 2, before the first episode of atrial fibrillation; they arose, therefore, in the perioperative period, when the patient was exposed both to microaxial pump support and to open cardiac surgery.

A right phrenic nerve palsy became apparent after extubation. Positive-pressure ventilation masks diaphragmatic paralysis, so its onset cannot be dated; it, together with pleural effusion, acute kidney injury, and infection, may have contributed to the protracted ventilatory course, and the timing of extubation therefore cannot be attributed to the pulmonary injury alone. It does not, however, account for the requirement for inhaled nitric oxide.

## Conclusions

A leaflet of a mitral bioprosthesis can tear away from a stent post 13 years after implantation and produce fulminant pulmonary edema within hours. When definitive surgery cannot begin immediately, the interval is not neutral: the lung continues to be injured, and, unlike the heart or the kidney, it has no durable artificial substitute and no realistic transplant option in this setting.

In this patient an Impella CP was inserted specifically to unload the left ventricle during a logistically imposed wait. Oxygenation was maintained and hemodynamics did not deteriorate during the five hours of support; the valve was replaced successfully; and the pulmonary infiltrates resolved almost completely by four weeks. The contribution of the device cannot be quantified from a single case. We suggest that when emergency surgery for acute left-sided valvular failure must be delayed, temporary left ventricular unloading deserves consideration as a means of protecting the lung. A single case can demonstrate feasibility but not optimal timing; whether the decision is better made when pulmonary congestion is first demonstrated than after the delay has become apparent or gas exchange has begun to fail is a hypothesis for comparative study rather than a management recommendation.
